# Pflegende Angehörige in Deutschland: Vereinbarkeit von Pflege und Erwerbstätigkeit

**DOI:** 10.1007/s00103-023-03687-3

**Published:** 2023-04-17

**Authors:** Adelheid Kuhlmey, Andrea Budnick

**Affiliations:** Institut für Medizinische Soziologie und Rehabilitationswissenschaft, Charité – Universitätsmedizin Berlin, Virchowweg 22, 10117 Berlin, Deutschland

**Keywords:** Belastung, Teilhabe, Fachkräftemangel, Familienpflegegeld, Work-Life-Balance, Burden, Participation, Labor shortage, Family care allowance, Work-life balance

## Abstract

Angehörige sind die tragende Säule der pflegerischen Versorgung in Deutschland. Knapp ein Viertel der Erwachsenen kennt eine hilfe- oder pflegebedürftige Person. Für immer mehr Menschen, unter ihnen mehrheitlich Frauen, wird die pflegerische Versorgung einer hilfebedürftigen Person zur alltäglichen Aufgabe. Diese Anforderung muss oft mit beruflichen Verpflichtungen und/oder der Erziehung minderjähriger Kinder vereinbart werden. Nicht nur in dieser „Sandwichposition“ vernachlässigen häuslich Pflegende eigene Lebensbereiche und gefährden ihre Gesundheit. Der narrative Übersichtsbeitrag fokussiert die Herausforderungen bei der Vereinbarkeit von häuslicher Pflege und Berufstätigkeit. Zudem wird die Bedeutung der Pflege durch Angehörige als relevantes Public-Health-Thema herausgearbeitet. Ein Spotlight wird auf die Versorgung pflegebedürftiger Kinder und die besonderen Ansprüche ihrer pflegenden Eltern gerichtet. Aktuelle Empfehlungen zur besseren Vereinbarkeit von Pflege und Beruf und zur Anerkennung der Sorgearbeit pflegender Angehöriger geben einen Ausblick auf Lösungsstrategien, die aus der Wissenschaft kommen und von der Politik aufgegriffen werden sollten.

## Abstract (en)

Informal caregivers are the main pillar of care provision in Germany. Almost a quarter of adults know a person who needs assistance or care. Caring for a person needing assistance is becoming an everyday task for more and more people. These demands must often be balanced with the requirements of work and/or care of underage children. Not only in this sandwich position informal caregivers neglect their own lives and endanger their health. The narrative review focuses on the challenges of reconciling care at home and work. In addition, the importance of informal caregiving as a relevant public health topic is discussed. A spotlight is placed on children in need of care and the particular demands of their caring parents. Current recommendations for a better reconciliation of care and work as well as for the recognition of relatives’ valuable caregiving work provide an outlook on solution strategies that come from science and should be addressed by policymakers.

## Einleitung

Die pflegerische Versorgung von hilfebedürftigen Personen wird für immer mehr Menschen in Deutschland, unter ihnen mehrheitlich Frauen, zur alltäglichen Aufgabe und muss oft mit beruflichen Verpflichtungen vereinbart werden. In dieser Doppelrolle werden häufig eigene Ansprüche an das Leben vernachlässigt – mit weitreichenden Folgen für die physische und psychische Gesundheit sowie die finanzielle und soziale Sicherheit. Der vorliegende Übersichtsbeitrag beantwortet die Fragen, welche Herausforderungen bei der Vereinbarkeit von häuslicher Pflege und Berufstätigkeit zu bewältigen sind und inwiefern dies ein relevantes Public-Health-Thema ist.

Zunächst werden Hintergrundinformationen zum Umfang und zu gesundheitlichen Auswirkungen der häuslichen Pflege in Deutschland gegeben. Danach werden Herausforderungen bei der Vereinbarkeit von häuslicher Pflege und Berufstätigkeit fokussiert und anschließend ein Spotlight auf die Versorgung pflegebedürftiger Kinder und die besonderen Ansprüche ihrer pflegenden Eltern gerichtet. In einem weiteren Abschnitt wird die Bedeutung der Pflege durch Angehörige als relevantes Public-Health-Thema herausgearbeitet. Zu guter Letzt werden aktuelle Empfehlungen vorgestellt, die die bessere Vereinbarkeit von Pflege und Beruf befördern sollen und eine Anerkennung der Sorgearbeit pflegender Angehöriger zum Ziel haben.

## Häusliche Pflege in Deutschland

**Figure 1:**
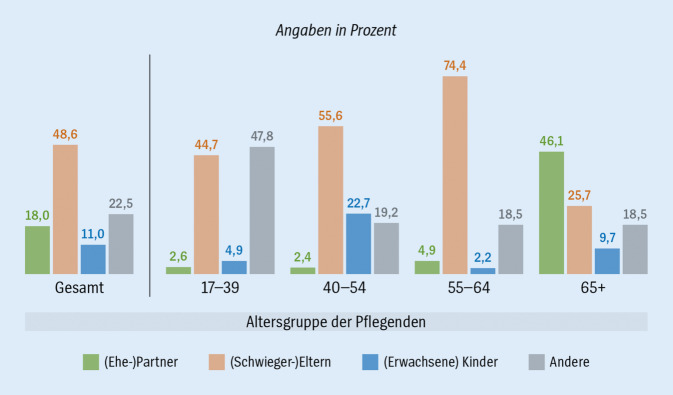

In Deutschland kennt knapp ein Viertel (24 %) der ab 17-Jährigen eine hilfe- oder pflegebedürftige Person. Tatsächlich übernehmen 9 % ab diesem Alter Hilfe- und Pflegetätigkeiten [1]. Der Vergleich zwischen pflegenden Personen und Nichtpflegepersonen zeigt auf der Basis von Daten der „Innovations-Stichprobe der Langzeiterhebung Sozio-oekonomisches Panel“ (SOEP-IS) im Jahr 2016 sowie des SOEP 2019, dass vorrangig ältere Menschen, eher Frauen und Verheiratete bzw. Personen mit Partner:in im Haushalt pflegen [1, 2]. Abb. 1 zeigt, dass im jüngeren Alter, vor allem aber im mittleren Erwachsenenalter (40–64 Jahre) Eltern und/oder Schwiegereltern gepflegt werden. Jüngere Erwachsene (17–39 Jahre) pflegen zu großen Teilen auch andere Personen (zum Beispiel Kinder). Pflegebedürftige Kinder und Jugendliche werden beinahe ausschließlich von ihren Eltern gepflegt, meistens von der Mutter [3]. Ab dem 65. Lebensjahr konzentriert sich die Angehörigenpflege eher auf (Ehe‑)Partner:innen [4].

Die Pflegestatistik zeigt, dass von aktuell 5,0 Mio. pflegebedürftigen Menschen in Deutschland die Mehrheit (4,17 Mio.) in der eigenen Häuslichkeit versorgt wird. Davon wiederum werden 2,55 Mio. ausschließlich durch Angehörige – zumeist Frauen – ohne professionellen Pflegedienst betreut [5]. Oftmals wird die häusliche Pflege von mehreren Personen ausgeübt, sodass von 3–5 Mio. pflegenden Angehörigen ausgegangen werden kann [6]. Pflegende Angehörige übernehmen diese Aufgabe häufig aufgrund einer emotionalen Bindung [7]. Die daraus entstehenden Risiken und Ressourcen der häuslichen Pflege für die Gesundheit der pflegenden Angehörigen sind seit mehr als 30 Jahren Gegenstand von Untersuchungen [8, 9]. Sie zeigen, dass Pflegende im Vergleich zu nicht pflegenden Personen eine vulnerable Gesundheit aufweisen, diese häufig vernachlässigen und vor allem durch fortwährende psychische Belastungen unter Erschöpfungszuständen leiden. Sie zeigen aber auch, dass die übernommene Sorgearbeit Ressourcen für ein gestärktes Selbstwertgefühl und ein Sinnerleben in sich birgt. Die Entscheidung für eine Pflegeübernahme wird neben den familialen Bindungsfaktoren auch durch kontextuelle, wohlfahrtsstaatliche und kulturelle Faktoren beeinflusst [10]. Eine sehr wichtige Determinante des Wohlbefindens häuslich Pflegender sind die autonome Motivation und Entscheidung zur Pflegeübernahme [11].

In Deutschland sind zwei Drittel aller pflegenden Angehörigen unter 65 Jahren erwerbstätig [6]. In Kombination mit einer beruflichen Beschäftigung können organisatorische, zeitliche und emotionale Herausforderungen der Pflegearbeit dazu führen, dass pflegende Angehörige die Arbeitszeit reduzieren oder ihre Tätigkeit ganz aufgeben. Eine Erwerbstätigkeit ist nicht per se eine Belastung für pflegende Angehörige, ein Belastungserleben tritt aber immer dann auf, wenn ein Vereinbarungskonflikt zwischen Arbeit und Pflege wahrgenommen wird [12]. Die Vereinbarkeit von Beruf und Pflege stand in den zurückliegenden Jahren zusätzlich im Spannungsfeld pandemiebedingter Herausforderungen (z. B. [13, 14]).

## Angehörigenpflege und Berufstätigkeit

**Figure 2:**
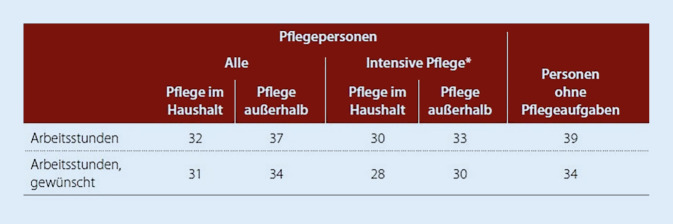

Von der Bevölkerung im Erwerbsalter (16–64 Jahre) pflegten 6 % einen Angehörigen [12].^1^ Die Angaben basieren auf Daten des Sozio-oekonomischen Panels (SOEP) aus den Jahren 2001–2012.^2^ Die Analysen des SOEP differenzieren zwischen Pflegepersonen, die mit einem pflegebedürftigen Menschen in einem Haushalt leben (Pflege im Haushalt), und denjenigen, die außerhalb des eigenen Haushaltes häusliche Pflege leisten (Pflege außerhalb, Abb. 2, 3 und 4), und zeigen Folgendes: Lebt die pflegebedürftige Person im Haushalt einer erwerbsfähigen Pflegeperson, sind es zu 57 % erwerbsfähige Frauen, die die häusliche Pflege übernehmen, während Männer sie lediglich zu 38 % ausüben. Lebt die pflegebedürftige Person im eigenen Haushalt (außerhalb des Haushaltes des Pflegenden) pflegen 5 % der erwerbsfähigen Personen zwischen dem 16. und 64. Lebensjahr [12]. Erwerbsfähige Pflegende sind vor allem Frauen und ältere Arbeitnehmer:innen. In den vergangenen 10 Jahren ist die Erwerbsbeteiligung dieser Gruppen am stärksten gestiegen [12]. Einschränkungen im Erwerbsleben treten bereits auf, wenn regelmäßig häusliche Pflegetätigkeiten von mehr als einer Stunde pro Tag ausgeübt werden. Analysen des SOEP zeigen zudem, dass pflegende Angehörige eine niedrigere Wochenarbeitszeit im Vergleich zu Personen ohne Pflegeaufgaben aufweisen (Abb. 2). Wird mehr als eine Stunde pro Tag gepflegt, liegt die wöchentliche Arbeitszeit bei 30 h, wenn die Pflege im selben Haushalt erfolgt (33 h bei Pflege außerhalb des eigenen Haushalts). Im Vergleich dazu sind Personen ohne Pflegeaufgaben 39 h beschäftigt. Zu einem ähnlichen Ergebnis kommt eine Untersuchung des Fraunhofer Instituts für angewandte Informationstechnik (FIT; 2021; [2]). Pflegende, die an Werktagen oder am Wochenende regelmäßig pflegen, sind rund 34 h pro Woche erwerbstätig. Bei Pflegenden, die nur an Werktagen regelmäßig pflegen, liegt der Erwerbsumfang rund 2 Stunden unter dem Vollzeitäquivalent [2].

**Figure 3:**
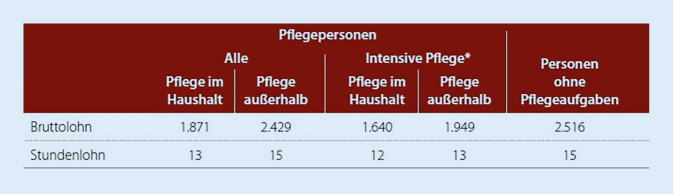

**Figure 4:**
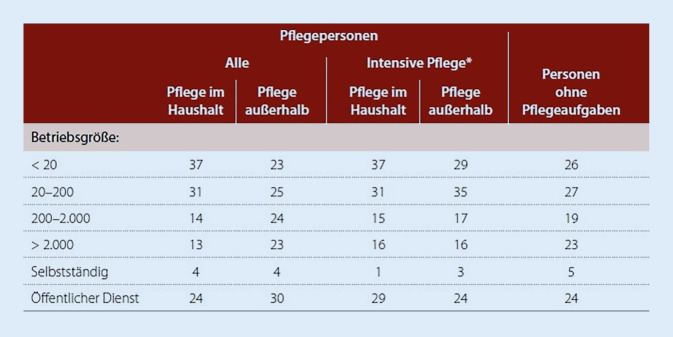

Die reduzierte Wochenarbeitszeit geht mit Einbußen im Erwerbseinkommen einher. Personen, die Pflegetätigkeiten im selben Haushalt übernehmen, verdienen 75 % (bei mehr als einer Stunde Pflege pro Tag nur 65 %) des Durchschnittseinkommens der Vergleichsgruppe ohne Pflegeaufgaben (Abb. 3). Pflegende, die außerhalb des eigenen Haushaltes und weniger als eine Stunde pro Tag pflegerische Aufgaben übernehmen, erzielen nahezu das Durchschnittseinkommen der Vergleichsgruppe ohne Pflegeaufgaben [12].

Die skizzierten Vereinbarkeitsprobleme von Pflege und Berufstätigkeit, welche auf Daten aus über einer Dekade (2001–2012) weit vor der COVID-19-Pandemie basieren [12], traten während der ersten Welle dieser Pandemie noch deutlicher zutage, weil Unterstützungsmöglichkeiten, wie z. B. die Tagespflege, entfielen. So gab ein Viertel der berufstätigen Pflegenden an, durch Arbeitszeitreduzierung oder mobiles Arbeiten Lücken der weggefallenen ambulanten Versorgung kompensiert zu haben [17]. Zusätzlich traten psychosoziale Belastungen auf. Zahlreiche Studien belegen physische und psychosoziale Auswirkungen der COVID-19-Pandemie auf pflegende Angehörige und arbeiten hierbei insbesondere die Verstärkung der Belastungssituation durch den Wegfall institutioneller Hilfen (z. B. die Schließung von Tagesbetreuungen) heraus. Als weitere Auswirkung zeigten sich bei den erwerbstätigen Pflegenden Ängste vor der Übertragung des Coronavirus auf die Pflegebedürftigen (z. B. [13, 14]).

In welchen Betrieben sind pflegende Angehörige beschäftigt? Sie sind häufig in Betrieben mit weniger als 20 Beschäftigten angestellt (Abb. 4). Das FIT differenziert dabei nach Geschlecht und verweist darauf, dass rund 28 % der erwerbstätigen pflegenden Frauen und rund 18 % der erwerbstätigen pflegenden Männer in Betrieben mit unter 20 Beschäftigten tätig sind [2]. Die Leistungen des Pflegezeitgesetzes (§ 3 PflegeZG) und des Familienpflegezeitgesetzes (§ 2 und 3 FPfZG) sind an die Größe eines Betriebes geknüpft. Ein Rechtsanspruch besteht erst ab 16 bzw. 26 Beschäftigten. In Betrieben mit mehr als 2000 Beschäftigten sind Pflegepersonen, die Pflege im eigenen Haushalt ausüben, unterrepräsentiert (Abb. 4). Selbstständige sind seltener Pflegepersonen im Vergleich zu Beschäftigten im öffentlichen Dienst [12].

Die vorangegangenen Ausführungen weisen auf Einschränkungen bei der Teilhabe am Arbeitsmarkt für erwerbsfähige Pflegepersonen im Vergleich zu erwerbsfähigen Nichtpflegenden hin. Der Ausstieg aus dem Erwerbsleben wird wahrscheinlicher, sobald die Vereinbarkeit von Pflege und Beruf schwieriger wird; dies trifft bereits zu, wenn der zeitliche Aufwand für die häusliche Pflege lediglich eine Stunde pro Tag beträgt [12, 16]. Dabei unterscheiden sich Frauen und Männer hinsichtlich ihrer Strategien, die häusliche Pflege zu bewältigen. Während erwerbstätige Frauen im Mittel eher eine Reduktion der Wochenarbeitszeit präferieren, ziehen sich Männer, insbesondere bei stärkerer Pflegebelastung, ganz aus dem Erwerbsleben zurück [12]. Berufstätigkeit kann einerseits negative Effekte häuslicher Pflegetätigkeit puffern [16], stellt aber andererseits hinsichtlich der Vereinbarkeit der beiden Tätigkeiten eine große Herausforderung dar, wie 71 % der pflegenden Beschäftigten angeben. Im beruflichen Alltag bedeutet dies neben versäumter Arbeitszeit, unterbrochenen Arbeitsabläufen oder Ängsten um den Verlust des Arbeitsplatzes auch Befürchtungen, Mobbing am Arbeitsplatz ausgesetzt zu sein [18].

Eine Studie der Antidiskriminierungsstelle des Bundes (2021) berichtet, dass knapp die Hälfte der Pflegenden (48 %) und knapp 2 Drittel der Eltern (64 %) Diskriminierungserfahrungen im Arbeitsleben im Kontext von Fürsorgeaufgaben, wie z. B. Elternschaft/Kinderbetreuung und -erziehung sowie Angehörigenpflege, machen [19].

Mit der demografischen Alterung schrumpft das Potenzial an pflegenden Angehörigen, während die Zahl pflegebedürftiger Menschen in unserer Gesellschaft steigt. Somit ist von einer zunehmenden Anzahl Erwerbstätiger auszugehen, die parallel zur Berufstätigkeit Hilfe- und Pflegetätigkeiten übernimmt. Eine bessere Vereinbarkeit von häuslicher Pflege und Berufstätigkeit kann nur gelingen, wenn professionelle, private und ehrenamtliche Unterstützungs- und Pflegeleistungen kombiniert werden und gesetzlichen Regelungen geschaffen werden, die auch Unternehmen in die Pflicht nehmen [6, 14, 20]. Insbesondere seitens der Arbeitgeber sind größere Anstrengungen dazu erforderlich. So bestätigten Personalentscheider mehrheitlich, dass Angebote zur Vereinbarkeit von häuslicher Pflege und Berufstätigkeit bisher weder vorhanden noch geplant sind. Berufstätige mit Pflegeerfahrung würden sogar vermeiden, über ein privates Pflegearrangement im beruflichen Umfeld zu sprechen, um berufliche Konsequenzen nicht zu evozieren [21]. Neuere Ergebnisse zeigen aber auch, dass sich knapp 2 Drittel der berufstätigen pflegenden Angehörigen (64 %) in der ersten Welle der COVID-19-Pandemie gut von ihrem Arbeitsgeber unterstützt fühlten [17]. Die Erwerbsbeteiligung von Unterstützungs- und Pflegepersonen blieb in der Pandemie stabil [22].

## Besonderheiten bei der Pflege pflegebedürftiger Kinder

Laut Pflegestatistik sind 160.953 Kinder und Jugendliche unter 15 Jahren pflegebedürftig (nach SGB XI). Dies entspricht in der Altersgruppe einer Pflegequote von 1,4 %. Die überwiegende Mehrheit (99,8 %) wird zu Hause versorgt. In vollstationären Einrichtungen werden 0,16 % (*n* = 268) der Kinder betreut [5]. Familien mit einem pflegebedürftigen Kind übernehmen in der Regel hauptverantwortlich die Pflege, welche individuell und in Abhängigkeit vom Krankheitsbild des Kindes heterogen, komplex und intensiv sein kann. Diese Aufgaben übernimmt vorrangig die Mutter (80 % der Fälle). Mit dem Partner teilen sich 17 % diese Aufgabe. Von den betroffenen Müttern beendet fast jede Vierte (26,2 %) in Deutschland ihr Arbeitsverhältnis. Jede Zweite (50,6 %) reduziert ihren Arbeitsumfang (vs. 21,8 % der Männer; [4, 22]). Somit tragen betroffene Mütter ein erhöhtes Risiko für Lücken im Erwerbsleben und für Armut, weil die Pflege der Kinder oft ein Leben lang notwendig ist [4, 23].

Die Diskrepanz zwischen der Wirklichkeit und dem Wunsch, gleichzeitig das eigene Kind zu versorgen und einer Berufstätigkeit nachzugehen, kommt in einem Befund des Instituts für Demoskopie in Allensbach zum Ausdruck: Lediglich 10 % der Eltern mit Kindern unter 6 Jahren befanden das Alleinverdienermodell (Mutter nicht berufstätig, Vater Vollzeit) noch als ideal [24]. Neben daraus resultierenden psychosozialen Herausforderungen, die betroffene Familien ab der Diagnosestellung bewältigen müssen, sind auch personelle Herausforderungen zu meistern [23].

Die Pflegeinfrastruktur für Kinder und Jugendliche ist in Deutschland noch unzureichend [25]. Neben dem allgemeinen Fachkräftemangel ist das professionelle Pflegepersonal nicht ausreichend für die Pflege von jungen Menschen spezialisiert. Ihre Pflege übernehmen häufig Kranken- oder Altenpfleger [26]. Ebenso blicken Erziehungs- und Lehrpersonal mit Sorge auf die hohe Verantwortung im Umgang mit pflegebedürftigen Kindern und Jugendlichen [27]. In der Forschung gewinnt das Thema hierzulande zunehmend an Bedeutung, wie jüngere Publikationen zeigen [28, 29]. Insgesamt bringt die Pflegebedürftigkeit aufgrund genetischer Beeinträchtigung, chronischer Erkrankung oder von Unfällen im Kindes- oder Jugendalter andere Anforderungen mit sich als die Pflege infolge von Alterungsprozessen [30]. Möglichkeiten, pflegebedürftige Kinder und Jugendliche sowie ihre Familien zu unterstützen, sind in gesellschaftlicher Teilhabe zu sehen. Diese kann u. a. durch die bessere Vereinbarkeit von Pflege und Beruf erreicht werden, wie die folgenden Abschnitte zeigen.

## Angehörigenpflege als ein relevantes Public-Health-Thema

Pflegende Angehörige versorgen deutlich mehr als die Hälfte der Pflegebedürftigen in Deutschland [5]. Sie sind damit eine zentrale Ressource im deutschen Pflegesystem. Durch den Personalmangel in der professionellen Pflege – nicht nur in der Altenpflege, sondern auch bei der Pflege von Kindern und Jugendlichen – wird die Relevanz pflegender Angehöriger als Leistungserbringer noch deutlicher. Eine Untersuchung des Zentrums für Qualität in der Pflege (ZQP) hat gezeigt, dass ambulante Pflegedienste nicht nur Neuanfragen von Unterstützungssuchenden ablehnten, sondern sie sogar Bestandskunden kündigten, da die Versorgung aufgrund von Personalmangel nicht hätte sichergestellt werden können [31]. Im Spannungsfeld zwischen Personalmangel im ambulanten Versorgungssetting und einer steigenden Zahl an pflegebedürftigen Menschen – vor allem aufgrund des Altwerdens der bevölkerungsstarken Generation der sog. Babyboomer – werden die Herausforderungen bezüglich der Vereinbarkeit von Angehörigenpflege und Erwerbstätigkeit zukünftig noch größer werden. Mit dem Ausscheiden der 13 Mio. Babyboomer aus dem aktiven Arbeitsleben bis 2030/2035 laufen wir nicht nur auf ein massives Problem bei der Versorgung dieser bevölkerungsstarken und alt werdenden Generation zu, es breitet sich zudem der Fachkräftemangel überall aus, der eben auch die professionelle Pflege betrifft. Ein Dilemma: Es fehlen die Pflegefachkräfte, die den Pflegebedürftigen und deren Angehörigen helfen könnten. Und die privat pflegenden Frauen und Männer werden zudem auf dem gesamten Arbeitsmarkt benötigt.

Diverse gesellschaftliche Veränderungsprozesse tragen zusätzlich zu der ungünstigen Entwicklung in der häuslichen Pflege bei. So nehmen berufliche und private Mobilität einen hohen Stellenwert in unserer Gesellschaft ein. Ebenso führen veränderte Familien- und Haushaltsstrukturen sowie die steigende Frauenerwerbsquote perspektivisch zur Erosion des Pflegepotenzials durch Angehörige [6]. Eine zunehmende Zahl an Singlehaushalten und kinderlosen Paaren führt diesen Trend deutlich vor Augen. Der Anteil an Singlehaushalten steigt seit den 1990er-Jahren in Deutschland kontinuierlich (1991: 11,9 Mio., 2000: 13,8 Mio. und 2016: 16,8 Mio.). Des Weiteren ist ein deutlicher Rückgang der Partnerschaften mit Kindern zu verzeichnen. Während 1996 noch die Hälfte der 25- bis 39-Jährigen in Partnerschaft und mit einem Kind lebte, waren es im Jahr 2016 nur noch ca. 37 % [32]. Zudem reduziert die zunehmende Frauenerwerbsquote (1960: 47 % im früheren Bundesgebiet, 2021: 76 % in Deutschland; [33, 34]) einerseits die objektive Verfügbarkeit und subjektiv möglichweise die Bereitschaft, Pflegetätigkeiten im Familien- und Angehörigenkreis zu übernehmen. Unabhängig von diesen sozialen Entwicklungen ist die Bereitschaft in den Familien groß, Verantwortung füreinander zu übernehmen (Solidaritätsprinzip; [35]). Dies ist ein weiterer wichtiger Grund, (Arbeits‑)Bedingungen zu schaffen, die die Vereinbarkeit von Angehörigenpflege und Berufstätigkeit ermöglichen. Sowohl aus gesellschaftlicher und politischer Perspektive als auch aus der Perspektive vieler Unternehmen besteht in Deutschland diesbezüglich nach wie vor Verbesserungsbedarf [21].

## Bessere Vereinbarkeit von Pflege und Beruf – aktuelle Empfehlungen für die Politik

Der unabhängige Beirat zur Vereinbarkeit von Pflege und Beruf^3^ legte im August 2022 im Rahmen der zweiten Berichtsperiode dem Bundesministerium für Familie, Senioren, Frauen und Jugend (BMFSFJ) „Empfehlungen zur Familienpflegezeit und zum Familienpflegegeld“ vor [36]. Ziel ist die Anerkennung der Arbeit pflegender Angehöriger und die Implementierung neuer sozialer Maßnahmen analog bestehender Unterstützungsleistungen für Eltern. Zentral ist dabei, dass die bisher geltenden Gesetze (PflegeZG und FPfZG) in ein Gesetz münden, welches sich analog den Regelungen zum Elterngeld und der Elternzeit in Regelungen für Freistellungen (Familienpflegezeit) und Regelungen für eine Entgeltersatzleistung (Familienpflegegeld) unterteilt. Die vom Beirat vorgeschlagenen Eckpunkte der neuen sozialen Maßnahme sollen pflegenden Angehörigen mehr Zeitsouveränität ermöglichen und langfristige finanzielle Nachteile durch den Lohnersatz verringern.

Dabei liegen die Limitationen der Vorschläge auf der Hand: Leider werden nicht alle Pflegenden gleichberechtigt in den Ausgleich einbezogen, was insbesondere in Vollzeit pflegende nichtberufstätige Personen betrifft, wie z. B. Mütter oder Angehörige von Personen mit Demenz. Eine generelle Bezahlung der Pflegearbeit würde diese Gruppen mitberücksichtigen. Dennoch sind die Empfehlungen zur Familienpflegezeit und zum Familienpflegegeld ein wichtiger Schritt für diejenigen, die Pflege und Beruf vereinbaren. Die Regelungen könnten ein Anreiz dafür sein, dass pflegende Mütter aus der Vollzeitpflegetätigkeit heraustreten und für die vollberufstätigen Väter ein Anlass, die Arbeitszeit zu reduzieren – so könnten beide Elternteile unter neuen gesetzlichen Rahmenbedingungen Pflege und Beruf vereinbaren, ohne zu große finanzielle Einbußen hinnehmen zu müssen.

In den Eckpunkten schlägt der Beirat des Weiteren vor, dass die Familienpflegezeit wahlweise als vollständige Freistellung für maximal 6 Monate oder als teilweise Freistellung genommen werden kann – insgesamt maximal 36 Monate. Die Zeitsouveränität der pflegenden Angehörigen soll auch erhöht werden, indem die Freistellung in mehrere Zeitabschnitte aufgeteilt werden kann. Das neue Familienpflegegeld soll ebenfalls für maximal 36 Monate bezahlt werden – die Höhe orientiert sich dabei am heutigen Elterngeld, denn Sorgearbeit soll als gleichwertig betrachtet werden. Darüber hinaus beinhalten die Beiratsvorschläge Verbesserungen der rentenrechtlichen Absicherung und sehen eine eigene Pflegezeit und ein eigenes Pflegegeld über einen Zeitraum von 3 Monaten für die Begleitung von Menschen in der letzten Lebensphase vor.

Und ein weiterer wichtiger Punkt: Der Kreis der Anspruchsberechtigten soll erweitert werden – künftig sollen nicht nur Angehörige, sondern alle vergleichbar nahestehenden Menschen einen Anspruch haben. Jeder der eine enge Beziehung zu einem pflegebedürftigen Menschen hat und Pflegeaufgaben übernehmen möchte, sollte darin unterstützt werden, Pflege und Beruf zu vereinbaren.

Geht es um weitere Anspruchsvoraussetzungen, wird für die Familienpflegezeit empfohlen, dass mindestens Pflegegrad 1 und für das Familienpflegegeld mindestens Pflegegrad 2 bei der pflegebedürftigen Person, die in der häuslichen Umgebung lebt, vorliegen muss. Bei minderjährigen pflegebedürftigen Personen kann die Pflege auch außerhäuslich stattfinden. Pflegende Angehörige müssen in einem Beschäftigungsverhältnis nach § 7 Absatz 1 PflegeZG stehen. Selbstständige werden ebenfalls berücksichtigt. Bei einer teilweisen Freistellung soll die wöchentliche Arbeitszeit max. 32 h betragen. Der Anspruch auf teilweise Freistellung besteht nicht in Unternehmen mit i. d. R. weniger als 15 Beschäftigten. Der Anspruch auf Familienpflegegeld sollte für die Zeit einer vollständigen Freistellung unabhängig von der Betriebsgröße bestehen und unter mehreren Angehörigen aufteilbar sein. Das steuerfinanzierte Familienpflegegeld sollte für Personen mit einem zu versteuernden Einkommen von mehr als 250.000 € pro Jahr nicht mehr bestehen. Das Einkommen der Pflegebedürftigen sollte nicht angerechnet werden [36].

Die Empfehlungen fließen in die Entwicklung einer Familienpflegezeit und einer staatlichen Lohnersatzleistung für Pflegende (Familienpflegegeld) ein, welche die Bundesregierung im Koalitionsvertrag 2021–2025 in Aussicht stellte und in den Blick nehmen wird [37, 38]. Dabei stellt sich die Frage, in welchem Umfang das Familienpflegegeld bei Einführung in Anspruch genommen werden würde. In einer aktuellen Mikrosimulation mit Daten des SOEP 2019 wird im Ergebnis von 1,9 Mio. Anspruchsberechtigten (bei mindestens 10 h häuslicher Pflege pro Woche) ausgegangen [39].

## Fazit

Der Beitrag zeigt die Bedeutung der Angehörigenpflege in unserer Gesellschaft auf. Er betont die Relevanz des Themas für die öffentliche Gesundheit in zweifacher Hinsicht: Einerseits sind pflegende Angehörige wichtige Leistungserbringer der Gesundheitsversorgung – das Bild von den Familien als größtem Pflegedienst Deutschlands findet hier den Rückhalt. Andererseits müssen pflegende Angehörige unter Public-Health-Perspektive auch als gesundheitlich besonders gefährdete Gruppe wahrgenommen werden. Die Ausführungen fassen zudem die Befundlage zur Vereinbarkeit von häuslicher Pflege und Berufstätigkeit zusammen und beschreiben die daraus entstehenden Herausforderungen für pflegende Angehörige, insbesondere auch für diejenigen die die Versorgung pflegebedürftiger Kinder übernommen haben.

Mit den Empfehlungen zur Familienpflegezeit und zum Familienpflegegeld soll die Vereinbarkeit von Pflege und Berufstätigkeit für häuslich Pflegende (Angehörige und andere nahestehende Personen mit Pflegeverantwortung) und pflegebedürftige Menschen in diversen familialen Netzwerken zukünftig verbessert werden. Ausgangspunkt dessen ist, dass die häusliche Pflege eine Alltagsaufgabe in unserer Gesellschaft geworden ist, die noch immer mehrheitlich von Frauen ausgeübt wird. Mit der Einführung einer Lohnersatzleistung für häuslich Pflegende soll u. a. eine Benachteiligung von Frauen möglichst vermieden werden – nicht nur hinsichtlich des Einkommens, sondern auch hinsichtlich des Erwerbs von Ansprüchen gegenüber der gesetzlichen Rentenversicherung. Damit werden Anreize für die partnerschaftliche Aufteilung der häuslichen Pflegearbeit gegeben. Dies könnte durch die Erweiterung des Kreises der Anspruchsberechtigten (nicht nur Angehörige, sondern alle vergleichbar nahestehenden Menschen) zusätzlich unterstützt werden. Letztendlich müssen die vorgestellten Empfehlungen von der Regierung aufgegriffen, in einen Gesetzesentwurf überführt und den weiteren Weg in die Gesetzgebung gehen, bevor pflegende Angehörige davon einen Nutzen haben.

## Footnotes

1 Die Aussagen basieren auf einer breiten Definition pflegender Angehöriger und umfassen diejenigen, die in ihrem sozialen Umfeld pflegebedürftige Menschen regelmäßig und nicht erwerbsmäßig unterstützen. Die Definition geht somit über die Definition nach §19 Elftes Buch Sozialgesetzbuch (SGB XI) hinaus, da auch Angehörige in die Analysen eingeschlossen sind, die einen hilfe- oder pflegebedürftigen Menschen versorgen, der den Kriterien für Leistungsbezieher:innen der Pflegeversicherung (noch) nicht entspricht [12, S. 27].

2 Anzumerken ist, dass die Datenlage zu erwerbstätigen Pflegepersonen derzeit noch lückenhaft ist und vermutlich wird die Zahl derjenigen, die von der Vereinbarkeitsproblematik betroffen sind, unterschätzt [15, 16].

3 Der unabhängige Beirat zur Vereinbarkeit von Pflege und Beruf wurde 2015 vom Bundesministerium für Familie, Senioren, Frauen und Jugend berufen, mit dem Ziel, das Pflegezeitgesetz und das Familienpflegezeitgesetz zu begleiten und sich mit Fragen der Vereinbarkeit zu befassen.
